# Effect of Empagliflozin on Left Ventricular Volumes in Patients With Type 2 Diabetes, or Prediabetes, and Heart Failure With Reduced Ejection Fraction (SUGAR-DM-HF)

**DOI:** 10.1161/CIRCULATIONAHA.120.052186

**Published:** 2020-11-13

**Authors:** Matthew M.Y. Lee, Katriona J.M. Brooksbank, Kirsty Wetherall, Kenneth Mangion, Giles Roditi, Ross T. Campbell, Colin Berry, Victor Chong, Liz Coyle, Kieran F. Docherty, John G. Dreisbach, Catherine Labinjoh, Ninian N. Lang, Vera Lennie, Alex McConnachie, Clare L. Murphy, Colin J. Petrie, John R. Petrie, Iain A. Speirits, Steven Sourbron, Paul Welsh, Rosemary Woodward, Aleksandra Radjenovic, Patrick B. Mark, John J.V. McMurray, Pardeep S. Jhund, Mark C. Petrie, Naveed Sattar

**Affiliations:** 1 Institute of Cardiovascular and Medical Sciences, British Heart Foundation Glasgow Cardiovascular Research Centre (M.M.Y.L., K.J.M.B., K.M., G.R., R.T.C., C.B., L.C., K.F.D., N.N.L., C.J.P., J.R.P., P.W., A.R., P.B.M., J.J.V.M., P.S.J., M.C.P., N.S.), University of Glasgow, United Kingdom.; 2 Robertson Centre for Biostatistics (K.W., A.M.), University of Glasgow, United Kingdom.; 3 Queen Elizabeth University Hospital, Glasgow, United Kingdom (M.M.Y.L., K.M., G.R., R.T.C., C.B., K.F.D., N.N.L., R.W., P.B.M., J.J.V.M., P.S.J.).; 4 Glasgow Royal Infirmary, United Kingdom (M.M.Y.L., G.R., J.R.P., M.C.P., N.S.).; 5 University Hospital Crosshouse, Kilmarnock, United Kingdom (V.C.).; 6 Golden Jubilee National Hospital, Glasgow, United Kingdom (R.T.C., C.B., J.G.D., M.C.P.).; 7 Forth Valley Royal Hospital, Larbert, United Kingdom (C.L.).; 8 University Hospital Ayr, United Kingdom (V.L.).; 9 Royal Alexandra Hospital, Paisley, United Kingdom (C.L.M.).; 10 University Hospital Monklands, Airdrie, United Kingdom (C.J.P.).; 11 West Glasgow Ambulatory Care Hospital, United Kingdom (I.A.S.).; 12 University of Sheffield, United Kingdom (S.S.).

**Keywords:** clinical trial, diabetes mellitus, empagliflozin, heart failure, magnetic resonance imaging, myocardium, sodium-glucose transporter 2 inhibitors, ventricular remodeling

## Abstract

**Background:**

Sodium-glucose cotransporter 2 inhibitors reduce the risk of heart failure hospitalization and cardiovascular death in patients with heart failure and reduced ejection fraction (HFrEF). However, their effects on cardiac structure and function in HFrEF are uncertain.

**Methods:**

We designed a multicenter, randomized, double-blind, placebo-controlled trial (the SUGAR-DM-HF trial [Studies of Empagliflozin and Its Cardiovascular, Renal and Metabolic Effects in Patients With Diabetes Mellitus, or Prediabetes, and Heart Failure]) to investigate the cardiac effects of empagliflozin in patients in New York Heart Association functional class II to IV with a left ventricular (LV) ejection fraction ≤40% and type 2 diabetes or prediabetes. Patients were randomly assigned 1:1 to empagliflozin 10 mg once daily or placebo, stratified by age (<65 and ≥65 years) and glycemic status (diabetes or prediabetes). The coprimary outcomes were change from baseline to 36 weeks in LV end-systolic volume indexed to body surface area and LV global longitudinal strain both measured using cardiovascular magnetic resonance. Secondary efficacy outcomes included other cardiovascular magnetic resonance measures (LV end-diastolic volume index, LV ejection fraction), diuretic intensification, symptoms (Kansas City Cardiomyopathy Questionnaire Total Symptom Score, 6-minute walk distance, B-lines on lung ultrasound, and biomarkers (including N-terminal pro-B-type natriuretic peptide).

**Results:**

From April 2018 to August 2019, 105 patients were randomly assigned: mean age 68.7 (SD, 11.1) years, 77 (73.3%) male, 82 (78.1%) diabetes and 23 (21.9%) prediabetes, mean LV ejection fraction 32.5% (9.8%), and 81 (77.1%) New York Heart Association II and 24 (22.9%) New York Heart Association III. Patients received standard treatment for HFrEF. In comparison with placebo, empagliflozin reduced LV end-systolic volume index by 6.0 (95% CI, –10.8 to –1.2) mL/m^2^ (*P*=0.015). There was no difference in LV global longitudinal strain. Empagliflozin reduced LV end-diastolic volume index by 8.2 (95% CI, –13.7 to –2.6) mL/m^2^ (*P*=0.0042) and reduced N-terminal pro-B-type natriuretic peptide by 28% (2%–47%), *P*=0.038. There were no between-group differences in other cardiovascular magnetic resonance measures, diuretic intensification, Kansas City Cardiomyopathy Questionnaire Total Symptom Score, 6-minute walk distance, or B-lines.

**Conclusions:**

The sodium-glucose cotransporter 2 inhibitor empagliflozin reduced LV volumes in patients with HFrEF and type 2 diabetes or prediabetes. Favorable reverse LV remodeling may be a mechanism by which sodium-glucose cotransporter 2 inhibitors reduce heart failure hospitalization and mortality in HFrEF.

**Registration:**

URL: https://www.clinicaltrials.gov. Unique identifier: NCT03485092.

## Abstract 2

Supplemental Digital Content is available in the text.

**Clinical Perspective**

**What Is New?**

- Although sodium-glucose cotransporter 2 inhibitors improve clinical outcomes in patients with heart failure with reduced ejection fraction, their effects on left ventricular remodeling are uncertain.
- To our knowledge, this is the first adequately powered cardiovascular magnetic resonance trial using a sodium-glucose cotransporter 2 inhibitor in patients with heart failure with reduced ejection fraction.
- In a total of 105 patients with heart failure with reduced ejection fraction and type 2 diabetes, or prediabetes, randomly assigned to empagliflozin 10 mg once daily or matching placebo and treated for 36 weeks, empagliflozin led to significant reductions in left ventricular end-systolic and end-diastolic indexed volumes of 6.0 and 8.2 mL/m^2^, respectively, and reductions in N-terminal pro-B-type natriuretic peptide by 28% (2%–47%), *P*=0.038.

**What Are the Clinical Implications?**

- Clinicians should be aware that the sodium-glucose cotransporter 2 inhibitor, empagliflozin, causes favorable cardiac remodeling in patients with heart failure with reduced ejection fraction.
- This may represent a mechanism by which sodium-glucose cotransporter 2 inhibitors reduce heart failure hospitalizations and cardiovascular death.

**Editorial, see p 526**

Adverse left ventricular remodeling, reflected by increased volumes and reduced contractility, is at the core of the pathophysiology of heart failure with reduced ejection fraction (HFrEF).^1,2^ Cardiac remodeling in HFrEF progresses over time, and larger left ventricular volumes are associated with worse nonfatal and fatal outcomes.^3–10^ Drug and device therapies that slow the progression of, or even reverse, remodeling are associated with better clinical outcomes in HFrEF. Sodium-glucose cotransporter 2 (SGLT2) inhibitors recently have been shown to reduce the risk of heart failure hospitalization and cardiovascular death in patients with HFrEF.^11,12^ The mechanism or mechanisms underlying these benefits are uncertain, including whether SGLT2 inhibitors have a favorable effect on cardiac remodeling. However, the reduction in NT-proBNP (N-terminal pro-B-type natriuretic peptide) observed with SGLT2 inhibitors is consistent with such an action.^11,12^ Empagliflozin has been shown to reduce left ventricular mass (indexed to body surface area) in people with type 2 diabetes and coronary artery disease, but the participants in this trial (the EMPA-HEART CardioLink-6 trial [Effects of Empagliflozin on Cardiac Structure in Patients with Type 2 Diabetes]) had normal left ventricular volumes and ejection fraction, and the decrease in left ventricular mass likely reflected reduction in weight and blood pressure, effects not associated with improved outcomes or favorable left ventricular remodeling in HFrEF.^13^ Only 1 prior trial has examined the effect of a SGLT2 inhibitor on left ventricular remodeling specifically in patients with HFrEF. The participants had relatively modest left ventricular enlargement and the trial was underpowered, with only 28 patients per treatment group.^14^ Consequently, we designed a larger, multicenter randomized, double-blind, placebo-controlled trial powered to investigate the effects of SGLT2 inhibition on left ventricular volumes in patients with HFrEF and type 2 diabetes or prediabetes.

## Methods

The data that support the findings of this study may be available from the corresponding author on reasonable request.

Fifteen hospitals in Scotland took part in the trial (details in the Data Supplement), each of which had ethical committee approval, and all participants gave written informed consent.

### Patients

Patients ≥18 years of age with HFrEF and type 2 diabetes (documented history of diabetes or previously undiagnosed diabetes with glycohemoglobin ≥48 mmol/mol [≥6.5%]), or prediabetes (glycohemoglobin 39–47 mmol/mol [5.7%–6.4%]) were potentially eligible. Participants had to be in New York Heart Association (NYHA) functional class II to IV and have a left ventricular ejection fraction (LVEF) ≤40%. The dose of angiotensin-converting enzyme inhibitor, angiotensin receptor blocker, or sacubitril/valsartan and β-blocker had to be unchanged for at least 4 weeks. Patients with type 2 diabetes were required to have a glycohemoglobin ≤97 mmol/mol (≤11%) and, if they were treated with glucose-lowering therapy, the treatment dose had to be unchanged for at least 6 weeks. The key exclusion criteria were type 1 diabetes, estimated glomerular filtration rate <30 mL/min/1.73m^2^, and history of diabetic ketoacidosis (a complete list of inclusion and exclusion criteria are provided in the Data Supplement).

### Randomization and Stratification

Patients were randomly assigned 1:1 to either empagliflozin 10 mg once daily or matching placebo by using an interactive web response system. The randomization sequence was performed in blocks of 4 and stratified by (1) age (<65 years, ≥65 years) and (2) diabetes or prediabetes status.

#### Schedule of Study Visits

Patients attended for 6 visits: screening, baseline, weeks 2, 12, 36, and 40 (Table I in the Data Supplement).

### Outcomes

#### Coprimary Outcomes

The coprimary outcomes were (1) left ventricular end-systolic volume indexed (LVESVi) to body surface area and (2) left ventricular global longitudinal strain (LV GLS). Between treatment group differences in the change from baseline to 36 weeks were calculated for both outcomes.

#### Secondary Outcomes

The secondary end points assessed at 36 weeks included additional cardiovascular magnetic resonance (CMR) measurements (body surface area–indexed left ventricular end-diastolic volume [LVEDVi], LVEF, body surface area–indexed left ventricular [LV] mass index, LV global function index, body surface area–indexed left atrial volume, myocardial blood flow, and extracellular volume fraction), diuretic intensification, assessment of symptoms using the Kansas City Cardiomyopathy Questionnaire Total Symptom Score (KCCQ-TSS), 6-minute walk distance, number of B-lines on lung ultrasound (a marker of lung congestion), and a range of biomarkers (as described in Biomarker Assessments and in the Data Supplement).^15–17^

### CMR Acquisition and Analysis

ECG-gated CMR was performed at 1 center (Queen Elizabeth University Hospital) at baseline prerandomization and week 36 using a 3.0 Tesla scanner (MAGNETOM Prisma, Siemens Healthcare). A week 40 scan after withdrawal of treatment was also planned but could not be performed in many patients because of the coronavirus disease 2019 (COVID-19) pandemic. The imaging protocol (Table II in the Data Supplement) included cine imaging, native T1 mapping (modified Look-Locker inversion-recovery), and delayed enhancement sequences. All scan acquisitions were spatially coregistered. CMR analysis methods are detailed in the Data Supplement.

### Biomarker Assessments

Glycohemoglobin, estimated glomerular filtration rate, uric acid, high-sensitivity cardiac troponin I, and galectin-3 (i1000SR, Abbott Laboratories, Abbott Diagnostics) were measured, along with NT-proBNP and growth differentiation factor-15 (e411, Roche Diagnostics) at baseline, 12 weeks, and 36 weeks. All of these assays were performed by using the manufacturers’ calibrators and quality controls.

### Statistical Analysis

Statistical analyses were conducted at the study data center (Robertson Center for Biostatistics, University of Glasgow) according to a prespecified Statistical Analysis Plan. All analyses were performed according to the intention-to-treat principle, including all randomly assigned participants with postrandomization data available for the outcome of interest at any given time point, irrespective of their subsequent participation in the study and their adherence to randomized treatment.

For all outcomes, analysis of covariance, with adjustment for randomized group, baseline value, and the stratification variables (age and diabetes/prediabetes status) were used to determine the between-group differences in the outcomes at 36 weeks. For the coprimary end points of LVESVi and LV GLS, the α was split so that a *P* value of 0.025 was considered statistically significant. No interim analyses were performed. For all secondary outcomes, a *P* value of 0.05 was considered statistically significant. All analyses were conducted using SAS version 9.4 (SAS Inc).

## Results

Recruitment took place between April 2018 and August 2019; follow-up visits were completed in May 2020. Of 166 patients screened, 105 were randomly assigned (52 to empagliflozin and 53 to placebo). Of these, 82 patients (78.1%) had diabetes and 23 (21.9%) had prediabetes.

### Baseline Characteristics

The mean (SD) age of participants was 68.7 (11.1) years, 77 patients (73.3%) were male, and 81 (77.1%) patients were in NYHA functional class II and 24 (22.9%) in NYHA class III. The median (interquartile range) duration of heart failure was 2.1 (1.0–4.8) years, and 52 patients (49.5%) had a history of heart failure hospitalization. Most patients had coronary artery disease (74 patients, 70.5%) and received standard heart failure therapy (Table 1). The mean (SD) CMR LVEF was 32.5% (9.8%), and median (interquartile range) NT-proBNP was 466 (177–1120) pg/mL.

**Table 1.**
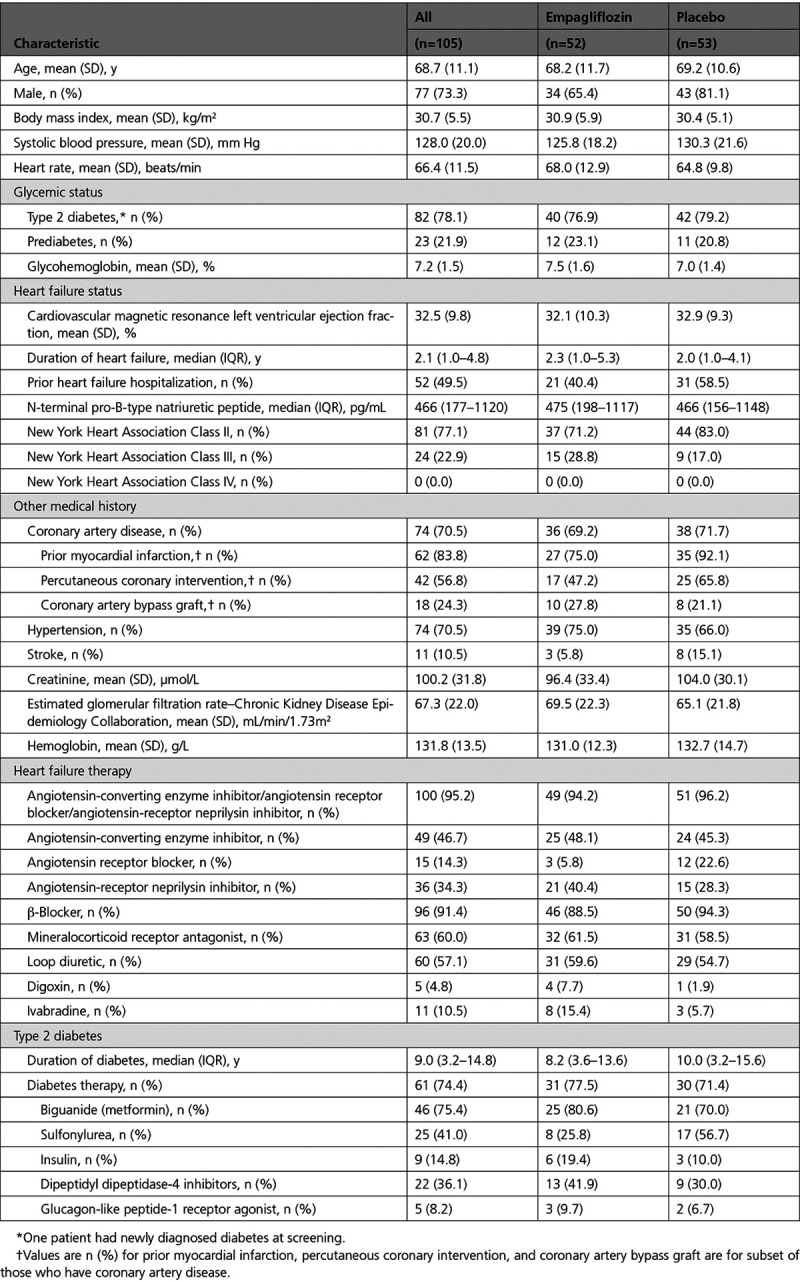
Baseline Characteristics of Randomized Patients

Diabetes treatments and other medical history are shown in Table 1.

### Completeness of Follow-Up and Adherence

Of the 52 patients randomly assigned to empagliflozin, 42 remained on randomized therapy and had complete coprimary end point data at baseline and week 36 (Figure I in the Data Supplement). Of the 53 patients randomly assigned to placebo, 50 remained on randomized therapy and had complete coprimary end point data at baseline and week 36. There were 2 deaths in the empagliflozin group, one attributable to newly diagnosed pancreatic cancer and one attributable to cardiogenic shock. There were no deaths in the placebo group.

### Coprimary Outcomes

**Figure 1.**
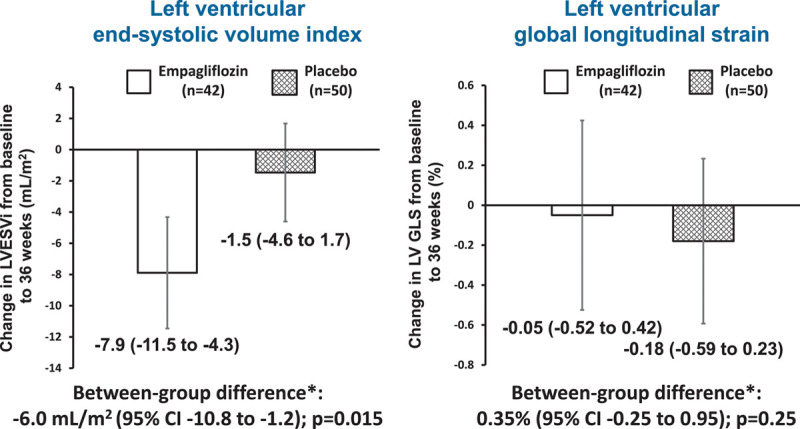
**Change in coprimary cardiovascular magnetic resonance outcomes from baseline to week 36.** Mean (95% CI). *Treatment effect calculated using an analysis of covariance model adjusted for treatment group, age at baseline, diabetes status, and baseline value. LVESVi indicates left ventricular end-systolic volume index; and LV GLS, left ventricular global longitudinal strain.

LVESVi decreased by 7.9 mL/m^2^ between baseline and 36 weeks in the empagliflozin group in comparison with a reduction of 1.5 mL/m^2^ in the placebo group: adjusted between-group difference –6.0 (95% CI, –10.8 to –1.2) mL/m^2^; *P*=0.015 (Table 2 and Figure 1). There was no difference in LV GLS between the empagliflozin and placebo groups: adjusted between-group difference 0.35% (95% CI, –0.25% to 0.95%); *P*=0.25. In view of potential imbalances in sex, NYHA class, and history of heart failure hospitalization at baseline, we did an additional post hoc adjustment for these variables that did not change our findings: adjusted between-group difference in LVESVi –5.9 (95% CI –11.0 to –0.9) mL/m^2^; *P*=0.023 and adjusted between-group difference in LV GLS 0.45% (95% CI, –0.17% to 1.08%); *P*=0.15.

**Table 2.**
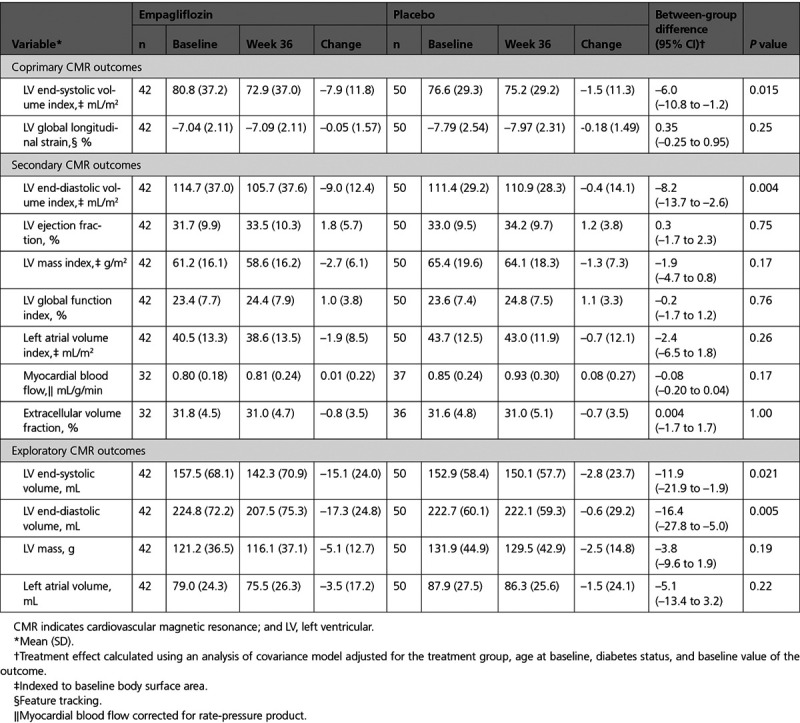
Change in CMR Parameters with Empagliflozin 10 mg/d or Placebo From Baseline to Week 36

### Secondary Outcomes

#### Cardiovascular Magnetic Resonance

**Figure 2.**
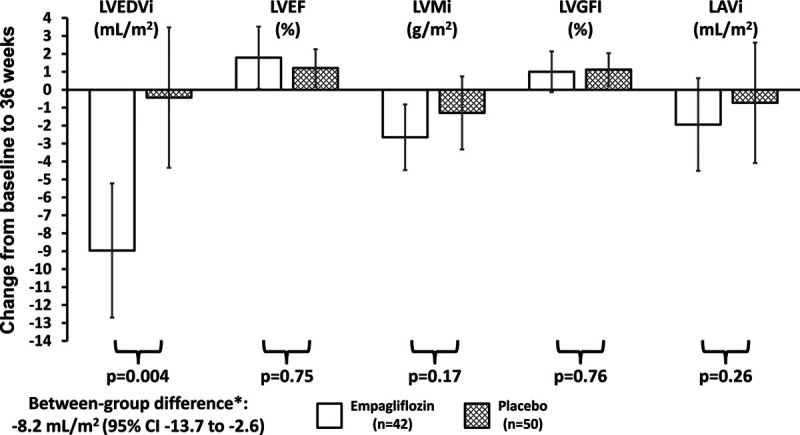
**Change in secondary cardiovascular magnetic resonance outcomes from baseline to week 36.** *Treatment effect calculated using an analysis of covariance model adjusted for treatment group, age at baseline, diabetes status, and baseline value. LAVi indicates left atrial volume index; LVEDVi, left ventricular end-diastolic volume index; LVEF, left ventricular ejection fraction; LVGFI, left ventricular global function index; and LVMi, left ventricular mass index.

LVEDVi decreased by 9.0 mL/m^2^ between baseline and 36 weeks in the empagliflozin group in comparison with a reduction of 0.4 mL/m^2^ in the placebo group: adjusted between-group difference –8.2 (–13.7 to –2.6) mL/m^2^; *P*=0.004. There were no significant changes in the other CMR variables (LVEF, LV mass index, LV global function index, indexed left atrial volume, myocardial blood flow, or extracellular volume fraction) after 36 weeks of treatment with empagliflozin, in comparison with placebo (Table 2 and Figure 2).

#### Diuretic Intensification, KCCQ-TSS, 6-Minute Walk Distance, and B Lines

There were no between-group differences in the intensification of diuretic therapy, change in KCCQ-TSS, 6-minute walk distance, or total number of B lines, between baseline and 36 weeks (Table 3).

**Table 3.**
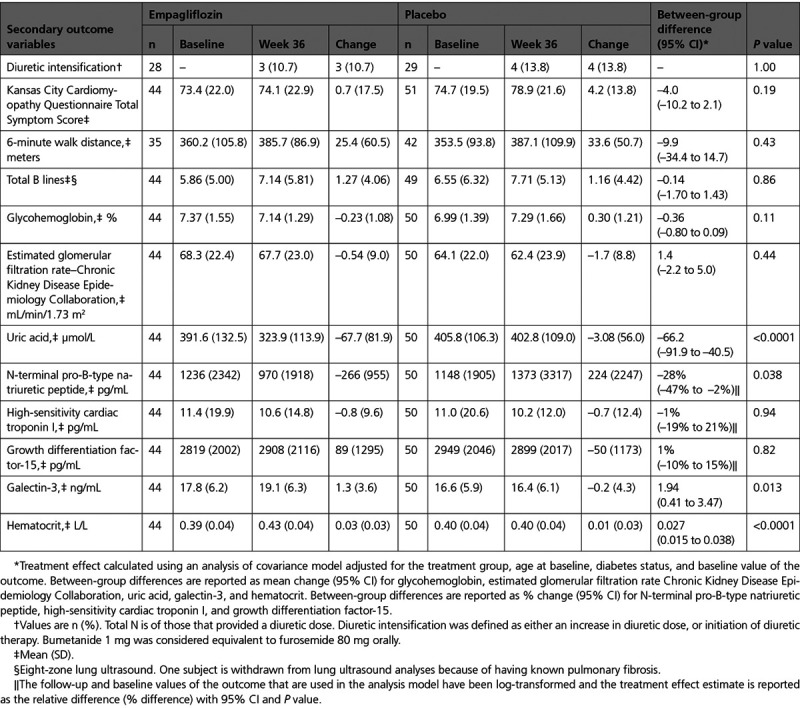
Change in Secondary Outcomes with Empagliflozin 10 mg/d or Placebo from Baseline to Week 36

#### Biomarkers

In comparison with placebo, empagliflozin reduced serum uric acid (*P*<0.0001) and NT-proBNP (*P*=0.038), and increased galectin-3 (*P*=0.013) and hematocrit (*P*<0.0001). There were no between-group differences in other biomarkers (Table 3).

### Safety and Exploratory Outcomes

There were no between-group differences in safety outcomes (Table III in the Data Supplement). Exploratory outcomes (blood pressure, heart rate, body weight, and blood ketones) are presented in Table IV in the Data Supplement.

## Discussion

We found that treatment of patients with HFrEF for 36 weeks with the SGLT2 inhibitor empagliflozin led to a significant reduction in LV volumes (LVESVi and LVEDVi) in comparison with placebo, but no improvement in LV GLS. Empagliflozin also reduced NT-proBNP. These favorable changes with empagliflozin were observed despite excellent conventional heart failure therapy, including sacubitril/valsartan in more than one-third of patients, and may explain, at least in part, the improvement in clinical outcomes observed with SGLT2 inhibitors in HFrEF. We did not observe an improvement in symptoms (using the KCCQ-TSS) or functional capacity (6-minute walk distance), although we had limited power to show a change in either of these measures. B-line number on lung ultrasound did not differ between treatment groups, and there was no difference in diuretic use, suggesting no major difference in surrogates of congestion between treatment groups.

Deleterious LV remodeling, including increases in end-diastolic and end-systolic volume and reduction in LVEF, is pathognomonic of HFrEF, and the extent of adverse remodeling correlates with risk of hospitalization and death.^1,2,18^ LV volumes and contractility worsen progressively over time, and slowing of this progression, or even reversal of adverse remodeling, is believed to be a key mechanism through which several pharmacological therapies improve clinical outcomes in HFrEF, as does cardiac resynchronization therapy. The magnitude of change in LV volumes observed at 36 weeks with empagliflozin compares favorably with the effects of other beneficial therapies in HFrEF and was incremental to those effects as our patients were comprehensively treated with renin-angiotensin system blockers and β-blockers. The more recently introduced therapies ivabradine and sacubitril/valsartan also reduced LV volumes when added to renin-angiotensin system blockers and β-blockers. In comparison with placebo, ivabradine reduced LVESVi by 5.8 mL/m^2^ and LVEDVi by 5.5 mL/m^2^ over 8 months in a substudy of SHIFT (Systolic Heart Failure Treatment With the *I*_f_ Inhibitor Ivabradine Trial).^5^ There have been 2 randomized trials of sacubitril/valsartan and remodeling. In the EVALUATE-HF trial (Effect of Sacubitril-Valsartan versus Enalapril on Aortic Stiffness in Patients With Heart Failure and Reduced Ejection Fraction), sacubitril/valsartan, in comparison with enalapril, reduced LVESVi by 1.6 mL/m^2^ and LVEDVi by 2.0 mL/m^2^ over 12 weeks.^6^ In the PRIME trial (Pharmacological Reduction of Functional, Ischemic Mitral Regurgitation), patients with an ejection fraction between 25% and <50% and mitral regurgitation were randomly assigned to sacubitril/valsartan or valsartan.^19^ The addition of neprilysin inhibition did not result in a reduction in LVESVi, but a reduction in LVEDVi (7 mL/m^2^) was observed. In SHIFT, EVALUATE-HF, and PRIME, LV remodeling was evaluated by using transthoracic echocardiography, but in the present study, we used the gold standard method of assessing cardiac structure and function with CMR.

We know of only 1 other trial examining the effect of a SGLT2 inhibitor on LV remodeling in patients with HFrEF. That trial, REFORM (Research Into the Effect of SGLT2 Inhibition on Left Ventricular Remodeling in Patients With Heart Failure and Diabetes Mellitus), did not show any improvement in LV remodeling with dapagliflozin.^14^ The likely explanations for the difference between the trials are that patients in REFORM had a higher LVEF (45.5% versus 32.5%), better NYHA class distribution (45% versus 0% NYHA class I), less LV enlargement (LVESVi 52 versus 77 mL/m^2^), and, especially, the much smaller number of randomly assigned (56 versus 105 patients), resulted in limited power to detect a difference between treatments.

A key remaining question is how does SGLT2 inhibition lead to favorable reverse remodeling? Empagliflozin may have reduced LV afterload, although the decreases in systolic and diastolic blood pressure were small and there was no significant reduction in LV mass, which might have been anticipated if there was an important and sustained reduction in afterload. Alternatively, SGLT2 inhibitors may cause a reduction in preload by inducing a diuresis; the consequent reduction in LV stretch may lead to a reduction in LV volumes. In the present study and in others, SGLT2 inhibitors have been shown to reduce levels of NT-proBNP, which is a surrogate marker for the degree of LV wall stress.^20^ The extent to which SGLT2 inhibitors exert a diuretic effect is debated, however, and we found no evidence of change in markers of pulmonary decongestion on lung ultrasound, no difference in conventional diuretic therapy, and no significant reduction in indexed left atrial volume. Alternatively, several additional mechanistic benefits of SGLT2 inhibitors on the myocardium have been proposed and these might also explain reduced LV volumes. A switch in myocardial energetics has been proposed but not proven in patients with HFrEF. SGLT2 inhibitors have also been proposed to reduce cardiac oxidative stress and inflammation through promotion of the actions of sirtuin-1 and upregulation of hypoxia-inducible factors signaling, but such hypotheses require confirmation.^21^ SGLT2 inhibitors do not appear to exert their actions by altering myocardial blood flow or extracellular volume, because neither was changed in the present trial.

We did not observe improvements in LV GLS with SGLT2 inhibition. Our patients had LV GLS values considerably below normal and consistent with those described in other studies in patients with HFrEF.^6,22^ As a relatively new measure of myocardial systolic function, the only other trial of LV remodeling in HFrEF to investigate LV GLS was the EVALUATE-HF trial.^6^ In comparison with enalapril, sacubitril/valsartan had no effect on LV GLS in that trial. Strain has not been reported in the older trials of LV remodeling, so it is unclear whether LV GLS is expected to change in parallel with LV volumes. We did not observe an increase in LVEF with empagliflozin treatment. In general, increases in LVEF have paralleled reductions in LV volumes in prior trials with β-blockers and ivabradine.^3–5^ It is unclear why we did not see an increase in LVEF, although this was not observed in the trials with sacubitril/valsartan mentioned earlier.^6,19^

### Limitations

We did not include patients with atrial fibrillation or patients with cardiac devices to avoid image degradation. We did not recruit any patients with NYHA functional class IV, although such patients were eligible. Treatment was given for only 36 weeks, and favorable remodeling in response to pharmacological therapy may continue over a longer period.^1^ Finally, in any modest-sized study such as this, imbalances in baseline characteristics can occur and potentially influence interpretation of the effects of randomized trials. To address this, we prespecified that we would adjust for the baseline value of the variable of interest, and factors used to stratify the randomization (age and diabetes status), as recommended in most guidelines for the analysis of clinical trials.

### Conclusion

In summary, treatment with the SGLT2 inhibitor empagliflozin led to favorable reverse LV remodeling in patients with HFrEF and type 2 diabetes or prediabetes. This finding may, at least in part, explain the beneficial effect of SGLT2 inhibitors on clinical outcomes in HFrEF.

## Sources of Funding

This trial was supported by an investigator-initiated study grant from Boehringer Ingelheim. Boehringer Ingelheim has provided support in terms of the funding and investigational medicinal product. The funder had no role in the design and conduct of the study; collection, management, analysis, and interpretation of the data; preparation or decision to submit the article for publication. Dr Berry, Dr McMurray, Dr M.C. Petrie, and Dr Sattar are supported by a British Heart Foundation Center of Research Excellence Grant (RE/18/6/34217). Dr Berry has received research support from the British Heart Foundation (PG/17/2532884, FS/17/26/32744, and RE/18/6134217) and the Medical Research Council (MR/S005714/1). Dr Mangion was supported by a British Heart Foundation Clinical Training Fellowship (FS/15/54/31639). Dr Sourbron was supported by the Innovative Medicines Initiative 2 Joint Undertaking (no. 115974, BEAt-DKD) and the Medical Research Council (MR/P023398/1, HEPARIM study [Hepatectomy Risk Assessment With Functional Magnetic Resonance Imaging]).

## Disclosures

Dr Lee’s employer, the University of Glasgow, has received grant support from Boehringer Ingelheim. Dr Berry is employed by the University of Glasgow, which holds consultancy and research agreements with companies that have commercial interests in the diagnosis and treatment of ischemic heart disease, including Abbott Vascular, AstraZeneca, Boehringer Ingelheim, GlaxoSmithKline, HeartFlow, Menarini Farmaceutica, Opsens, Philips, and Siemens Healthcare. Dr Docherty’s employer, the University of Glasgow, is paid by AstraZeneca for involvement in the DAPA-HF trial. Dr J.R. Petrie has received research grants from Juvenile Diabetes Research Foundation. He has also received personal fees and travel support from Novo Nordisk, research grants and personal fees from Janssen, personal fees from Abbott, ACI Clinical, Biocon, and Iqvia, and nonfinancial support from AstraZeneca, Dexcom, Merck KGaA (Germany), and Itamar Medical. Dr Sourbron reports research funding from European Federation of Pharmaceutical Industries and Associations (EFPIA) through the Innovative Medicines Initiative, and from Bayer AG, GlaxoSmithKline, and General Electric for cofunded PhD studentships. Dr Mark reports research funding from Boehringer Ingelheim, paid advisory boards from AstraZeneca and Vifor-Fresenius, lecture fees from Novartis, Pfizer, and Bristol Myers Squibb, and travel support from Pharmacosmos. Dr McMurray’s employer, the University of Glasgow, has been paid by AbbVie, Amgen, AstraZeneca, Bayer, Bristol Myers Squibb, DalCor, GSK, Merck Sharp & Dohme, Novartis, Resverlogix, and Theracos for his participation in clinical trials and by Alnylam, AstraZeneca, Cardurion, Novartis, and Pfizer for consultancy, advisory board membership, or lectures. Dr Jhund’s employer, the University of Glasgow, is paid by AstraZeneca for involvement in the DAPA-HF (Study to Evaluate the Effect of Dapagliflozin on the Incidence of Worsening Heart Failure or Cardiovascular Death in Patients with Chronic Heart Failure) and DELIVER (Dapagliflozin Evaluation to Improve the Lives of Patients With Preserved Ejection Fraction Heart Failure) trials. He has also received consulting, advisory board, and speaker’s fees from Novartis and AstraZeneca, advisory board fees from Cytokinetics, and a grant from Boehringer Ingelheim. Dr M.C. Petrie has received research grants or consultancy fees from SQ Innovations, AstraZeneca, Roche, Boehringer Ingelheim, Eli Lilly, Napp Pharmaceuticals, Novartis, and Novo Nordisk and has served on clinical events committees for AbbVie, Alnylam, AstraZeneca, Bayer, Boehringer Ingelheim, GlaxoSmithKline, Resverlogix, and Novo Nordisk. Dr Sattar has consulted for or received lecture fees from Amgen, AstraZeneca, Boehringer Ingelheim, Eli Lilly, Merck Sharp & Dohme, Novartis, Novo Nordisk, Pfizer, and Sanofi. He has received grant support from Boehringer Ingelheim through his institution, the University of Glasgow.

## Supplemental Materials

Data Supplement Methods

Data Supplement Results

Data Supplement Tables I–IV

Data Supplement Figure I

References 23–32

## Supplementary Material


