# Supplementary material for: Effect of Empagliflozin on Left Ventricular Volumes in Patients With Type 2 Diabetes, or Prediabetes, and Heart Failure With Reduced Ejection Fraction (SUGAR-DM-HF)
Source: Circulation. 2020 Nov 13;143(6):516–25. doi: 10.1161/CIRCULATIONAHA.120.052186 (PMC7864599; doi:10.1161/CIRCULATIONAHA.120.052186)
Supplement: Supplementary file 1 [file cir-143-0516-s001.pdf]

Effect of empagliflozin on left ventricular volumes in patients with type 2 diabetes, or prediabetes, and heart failure with reduced ejection fraction (SUGAR-DM-HF)

SUPPLEMENTAL MATERIAL

Supplemental Methods

Study Setting

Patients were recruited from a total of 15 hospitals in Scotland, United Kingdom: 2 large urban hospitals (Queen Elizabeth University Hospital, Glasgow Royal Infirmary), 3 ambulatory care hospitals (Stobhill Ambulatory Care Hospital, West Glasgow Ambulatory Care Hospital, New Victoria Hospital) and 10 regional hospitals (Inverclyde Royal Hospital, Vale of Leven Hospital, Royal Alexandra Hospital, Golden Jubilee National Hospital, University Hospital Monklands, University Hospital Hairmyres, University Hospital Wishaw, Forth Valley Royal Hospital, Crosshouse Hospital, University Hospital Ayr).

Patients

Eligibility for randomization in the trial was based on the following criteria:

Inclusion Criteria:

1. age ≥18 years
2. heart failure (HF) with left ventricular ejection fraction (LVEF) ≤40% on screening visit echocardiogram, New York Heart Association (NYHA) II-IV symptoms, stable doses of angiotensin-converting enzyme inhibitor, angiotensin receptor blocker, angiotensin-receptor neprilysin inhibitor or beta-blocker for 4 weeks prior
3. type 2 diabetes (HbA1c ≤97 mmol/mol (≤11%), diet-controlled or on stable treatment for 6 weeks prior) or prediabetes (HbA1c 39-47 mmol/mol (5.7-6.4%))

Exclusion Criteria:

1. type 1 diabetes
2. diabetic ketoacidosis
3. insulin use within 1 year of diagnosis of diabetes
4. history of acute or chronic pancreatitis and on insulin treatment for diabetes or low residual c-peptide (random non-fasting level of <0.2 nmol/l
5. eGFR <30 ml/min/m^2 (based on latest available result)
6. persistent/permanent atrial fibrillation/flutter
7. acute coronary syndrome, stroke or surgery within 1 month
8. body mass index >52 kg/m^2
9. liver disease (defined by serum alanine transferase, aspartate aminotransferase, alkaline phosphatase >3x upper limit of normal)
10. bariatric surgery within the past two years and other gastrointestinal surgeries that induce chronic malabsorption
11. any condition with life expectancy <2 years
12. active malignancy requiring treatment (except successfully treated basal cell or squamous cell carcinoma, adjuvant hormonal therapy for breast or prostate cancer
13. blood dyscrasias or any disorders causing hemolysis or unstable red blood cells
14. systemic steroids within 6 weeks prior
15. any uncontrolled endocrine disorder except type 2 diabetes or prediabetes

16. alcohol/drug abuse within 3 months that would interfere with trial participation or any ongoing condition leading to decreased compliance with study procedures or study drug intake
17. known hypersensitivity to empagliflozin or excipients
18. known hypersensitivity to gadolinium
19. inability to give informed consent
20. sodium-glucose cotransporter 2 (SGLT2) inhibitor use (current or previous)
21. devices or any other contra-indication to magnetic resonance imaging (MRI)
22. currently pregnant, planning pregnancy, or currently breastfeeding
23. history of previous lower limb amputation (non-traumatic)
24. current participation in another interventional medical study or within the last 90 days
25. anyone who, in the investigator's opinion, is not suitable to participate in the trial for other reasons (e.g. claustrophobia)

Outcomes

Co-Primary Efficacy Outcomes

1. left ventricular end-systolic indexed to body surface area (LVESVi) (cardiovascular magnetic resonance (CMR))
2. left ventricular global longitudinal strain (GLS) (CMR feature-tracking)

Secondary Efficacy Outcomes

1. left ventricular end-diastolic volume indexed to body surface area (LVEDVi) (CMR)
2. left ventricular ejection fraction (LVEF) (CMR)
3. left ventricular mass indexed to body surface area (LVMi) (CMR)
4. left ventricular global function index (LVGFi) (CMR)
5. left atrial volume indexed to body surface area (LAVi) (CMR)
6. myocardial microvascular perfusion (contrast-enhanced CMR myocardial blood flow (MBF))
7. extracellular volume fraction (ECV) (contrast-enhanced CMR)
8. intensification of diuretic therapy
9. quality of life (Kansas City Cardiomyopathy Questionnaire (KCCQ) – Total Symptom Score (TSS) (mean overall difference and responder analysis))
10. exercise capacity (six minute walk distance)
11. pulmonary congestion (B-lines measured using lung ultrasound)
12. biomarker profile including Plasma glycated hemoglobin (HbA1c), creatinine, estimated glomerular filtration rate chronic kidney disease epidemiology collaboration (eGFR CKD-EPI), liver function tests, uric acid, troponin I, N-terminal pro-B-type natriuretic peptide (NT-proBNP), growth differentiation factor-15 (GDF-15), and Galectin-3

Safety Outcomes

1. hepatic injury
2. renal dysfunction / hyperkalemia / metabolic acidosis
3. proportion with serum creatinine > 2.5 mg/dl (>221 micromol/l)
4. ketoacidosis

Stratification and Capping

Recruitment was continuously monitored in order to achieve adequate and representative proportions of patient sub-populations with diabetes and prediabetes. Prediabetes populations

were capped at 40%, in order to ensure approximate balance between treatment groups within each sub-population.

Screening

The clinical research team on each site screened patients in primary and secondary care. Patients aged ≥18 years, of either sex, with a history of HF and type 2 diabetes or prediabetes were prospectively identified, supported by review of (electronic) health records. Potential participants were approached (and given patient information sheet) either in person by the clinical care team at a routine outpatient appointment, or by letter drop. Interested patients were then followed up by telephone to ascertain interest and confirm initial eligibility, and then invited to attend for an initial screening visit. If device therapy was indicated or further up titration of heart failure and/or diabetes therapy was required, screening was put on hold.

Screening and Rescreening

All patients attended an initial screening visit for an echocardiogram, and if required updated blood tests for eGFR, liver function tests and HbA1c. This initial screening helped determine eligibility prior to the baseline visit. There was the option of combining the screening visit with the baseline visit to minimize imposition on patients. Patients who initially failed screening could be re-screened once more. To minimize patient burden, patients could be rescreened using the result from the previous screening echocardiogram (if performed within the last 12 weeks, as long as there was no clinical reason to suspect a change in LVEF). For the purposes of determining eligibility, in the absence of a history of liver disease, liver function tests did not specifically need to be repeated at the screening visit (if the screening visit is separate to the baseline visit). For the purposes of determining eligibility, an eGFR value of ≥30 ml/min/1.73m^2 at the screening visit (if the screening visit is separate to the baseline visit) was deemed satisfactory to allow randomization on the baseline visit (before the result of the baseline eGFR was available). This minimizes imposition of patients and prevents delays in randomizing patients (with subsequent delays in issuing of prescriptions to patients) at the baseline visit.

CMR Acquisition and Analysis

Observers were blinded to subject ID, date and time of scan, clinical data and randomization arm. All analyses were performed in a paired fashion (i.e. the baseline and follow-up were examined one after another) to minimize variability. All analyses were performed by a single experienced (>4 years) observer (M.M.Y.L.), adhering to pre-specified standard operating procedures (SOPs). Inter-observer analyses were performed by an experienced (>7 years) observer (K.M.) for 10% of scans, which were selected at random. A Likert scale was used to assess image quality for each component (1 = good or no major artefact; 2 = adequate or only minor artefact; 3 = poor or major artefact). Scans with a Likert scale of 3 were not analyzed. For clinical imaging governance purposes, all CMR studies were prospectively reported (by G.R.) (separate from study analyses) including for incidental non-cardiac findings.

Volumetric Analysis

Analysis of ventricular volumes, feature-tracking GLS, and ECV was performed using commercially available software cvi42 version 5.9.4 (Circle Cardiovascular Imaging, Canada) following a standard protocol taught by the software manufacturer.
Left ventricular (LV) volumes and mass were analyzed using techniques previously described.[23] LV papillary muscles and trabeculations were excluded from calculations of LV mass and included as part of the ventricular blood pool. The subaortic LV outflow tract was

included as part of the blood pool. Internal quality control steps included checking for consistency between: (i) LV mass measured in end-diastole and end-systole; (ii) LV and right ventricular stroke volumes (taking into account reasons for potential discrepancies e.g. valvular regurgitation, shunts); (iii) the most basal LV slice selection at baseline and follow-up scans.
Maximal LAV was calculated with the biplane area-length method from manually drawn LA endocardial contours in the end-systolic phase (maximal LA area).[23] Pulmonary veins and left atrial appendage were excluded from LAV.[24]

GLS
Feature-tracking strain analysis was quantified based on manually contoured LV endocardial and epicardial borders from the short-axis stack and three long-axis (2-, 3- and 4-chamber) cine images in the LV end-diastolic phase (the reference phase). The performance of myocardial tracking was visually checked in case of insufficient tracking, and contours readjusted as necessary and the tracking algorithm rerun. LV GLS was calculated from the average of the peak strain of the 3 long axis slices.[25,26]

LVGFI
LVGFI is a marker integrating cardiac structure and function, with evidence supporting its prognostic value.[27-29] LVGFI = (LV stroke volume/LV global volume) * 100, where stroke volume is calculated by LVEDV – LVESV, and LV global volume as the sum of the LV mean cavity volume ([LVEDV+ LVESV/2]) and the myocardial volume (LV mass/specific myocardial density coefficient 1.05 g/ml).

Contrast-Enhanced MRI
A single mid-LV myocardial dynamic-contrast enhanced (DCE) CMR was performed at baseline and 36 weeks. The gadolinium contrast used was Gadovist (Gadobutrol, strength 1.0 M solution, Bayer PLC). A contrast bolus of 0.025 mmol/kg was used to quantify MBF. A total contrast dose of 0.15 mmol/kg was used to quantify ECV. If renal function was impaired with eGFR <30 ml/min/1.73m^2 at the time of week 36 MRI scan, gadolinium contrast was not used and therefore secondary outcomes related to contrast enhancement (MBF, ECV) were not obtained.

MBF
Quantitative cardiac perfusion (MBF at rest) was analyzed with open source software PMI-0.4, as described in our SOP (available online).[30] Image pre-processing, segmentation and motion correction were performed prior to pharmacokinetic modelling. The number of dynamics were cropped at 60 seconds after the time of contrast first arriving in LV blood pool, in order to minimize the impact of poor motion correction in later dynamics. A myocardial region of interest (ROI) was manually drawn on motion-corrected cardiac DCE images. An arterial input function (AIF) ROI was drawn within LV blood pool. The quality of motion correction was assessed, and contours adjusted as required to avoid partial volume effects from neighboring structures and blood pool. Cardiac DCE was modelled using a 1-compartment model to calculate myocardial blood flow (MBF), using a hematocrit taken on the same day. MBF values were corrected for rate-pressure product with the formula = MBF * (10000/RPP), where RPP is Rate Pressure Product (systolic blood pressure SBP * heart rate).

ECV

Color-coded T1 and ECV maps were generated based on inline-generated, motion corrected raw images. Global myocardial ECV was analyzed by manually contouring LV endocardial and epicardial myocardium and LV blood pool in a single short axis mid-LV slice in both pre- and post-contrast T1 maps. An endocardial and endocardial offset of 20% was used to avoid partial volume effects from neighboring structures and blood pool. Global ECV was then calculated from pre- (native) and post-contrast myocardial and blood pool T1 values, together with a hematocrit taken on the same day.[31]

Assessing MRI Tolerability Before Randomization to Minimize Dropouts

Patients who completed the baseline MRI measurement required for the primary endpoint were randomized. Patients who did not complete the baseline MRI measurement required for the primary endpoint were not randomized, but were classed as screen failures. Therefore, this did not affect patient safety, eligibility, trial integrity or the primary endpoint.

Lung Ultrasound

Lung ultrasound was performed at 4 visits (baseline, weeks 12, 36 and 40), using techniques previously described.[17] A SIEMENS Acuson SC2000 machine and transducer phased array probe was used. The examination was performed on eight zones (four zones on each hemi-thorax). Patients were positioned semi-recumbent at 45 degrees. Six second clips were recorded. Scan depth was set at 18 centimeters. The maximum number of B-lines in any still frame was counted for each zone, and the total number of B-lines per patient calculated as the sum of each zone.

Withdrawal and Discontinuation Criteria, Safety Questions

In case of acute illness or conditions leading to fluid loss, patients were advised to temporarily discontinue study drug, as well as to seek urgent medical advice. All participants were provided with a study alert card, with details of emergency unblinding arrangements and signs of diabetic ketoacidosis (DKA). An additional information sheet for participants also provided advice on “sick day rules”. A 24/7 study mobile number was provided to all patients.

Subjects were free to stop participating at any point during the trial but were encouraged to remain under trial follow-up if they opted to discontinue study drug. However, they were able to withdraw consent for any further participation.

For patients who prematurely discontinue study treatment before at least 3 months of cumulative drug exposure, we decided to not perform all planned follow-up assessments (such as MRI).

For patients who prematurely discontinued study treatment after at least 3 months of drug exposure, we will perform week 36 follow-up assessments (including MRI) as soon as possible, and ideally before stopping study treatment, if patients agreed to continue participation.

If the answer to any of the safety questions below:

1. Any symptoms of hypotension/volume depletion including presyncope/syncope and falls (and measured blood pressure)
2. Any urinary tract infection episodes

3. Any genital infections
4. Renal dysfunction / Hyperkalemia / Metabolic acidosis (complemented by study visit biochemistry)
5. Ketoacidosis signs and symptoms as specified in our patient alert card (complemented by study visit biochemistry)
6. Hepatic injury (complemented by study visit biochemistry to check for Hy’s law)
7. Lower limb amputation

at any visit is Yes, we will:

- Assess the need for discontinuation of study medication:
  o #1,2,3 – May not require discontinuation
  o # 4, 5 – At least temporary discontinuation (if DKA confirmed by biochemistry, then permanent discontinuation)
  o #6,7 – Permanent discontinuation
- Consideration should also be given to stopping study medication if patients develop foot complications such as infection, skin ulcers, osteomyelitis, or gangrene
- Schedule an additional study visit to check bloods and assess possibility of restarting study medication with the patient’s full permission and close monitoring for the AE that triggered the extra study visit.

The study doctor will discontinue investigational medicinal product (IMP) in any participant who develops any of the following during the study:

- Pregnancy
- eGFR $\leq$20 mL/min/m^2 on two consecutive blood samples. The rationale for the choice of this cut-off is explained in protocol section 2.1.1
- Confirmed symptomatic hypoglycemia (plasma glucose <2.5 mmol/L) that cannot be rectified by alteration of other background antidiabetic agents. This is supported by initial glycemic control inclusion criteria
- Hypersensitivity to IMP
- Confirmed diagnosis of DKA
- Confirmed diagnosis of Fournier’s gangrene (necrotizing fasciitis of the perineum)
- Subsequent contra-indication to MRI e.g. clinical need for implantable cardiac device insertion (e.g. permanent pacemaker, implantable cardioverter defibrillator, cardiac resynchronization therapy)

Additional study visits were scheduled as clinically indicated, even if not meeting adverse event / serious adverse event / adverse event of special interest criteria. Alternatively, patients’ routine clinical care team (with their prior agreement) may be asked to update clinically indicated blood tests, if patients prefer this, in order to minimize inconvenience.

Follow-Up and Timing of Outcome Evaluations

Following randomization, clinical assessments involved gathering information from the standard-of-care clinical reviews, and also from clinical contacts recorded in the patients’ medical records. In West of Scotland hospitals, a single system of electronic patient records is used for all hospital attendances and correspondence with primary care.

Impact of Coronavirus Disease 2019 (COVID-19) on Timelines

In response to the COVID-2019 pandemic in March 2020, a small number n=7 of week 36 study visits were scanned earlier (between 1-3 weeks earlier), and this was approved by the sponsor regulatory authorities. One patient did not attend the week 36 study visit due to the

pandemic restrictions. The remainder n=21 of remaining week 40 study visits were cancelled but completed by phone call instead.

Data Management and Biostatistics

The Robertson Centre for Biostatistics acted as an independent coordinating center for randomization, data management and statistical analyses. The Centre is part of the registered Glasgow Clinical Trials Unit (National Institute for Health Research (NIHR) Registration number: 16).

Statistical Analysis

We calculated that with 40 patients per group, the trial would have >80% power to detect a 6 ml/m^2 difference in LVESVi (10 ml difference in non-indexed LVESV) assuming a standard deviation (SD) of ≤8.4 ml/m^2 (SD of LVESV ≤14 ml), and a 5% difference in myocardial strain (global longitudinal), assuming a SD of ≤7%. These differences are generally considered to be clinically meaningful differences.[2,18,22,32] A Statistical analysis plan was finalized prior to unblinding. All analyses were conducted according to the intention to treat (ITT) principle and will use regression analysis models that are adjusted for the randomization stratification variables, age and diabetes/prediabetes status. We did not impute missing data. All CMR measurements were indexed to baseline body surface area (BSA), derived using the Mosteller formula. Patients who were in atrial fibrillation or atrial flutter at the time of the week 36 visit were excluded from the primary analysis, but remained in the randomized population.

Trial Management

Trial Steering Committee (TSC): A TSC monitored the design and conduct of the trial and made recommendations to the CI and Sponsor on the trial conduct and on modifications to the protocol and/or trial procedures. The TSC consists of an independent chairperson (R.L.), two independent members (R.M., R.P.) and representatives of the Sponsors (NHS Greater Glasgow and Clyde (NHS GG&C) and the University of Glasgow).

Trial Management Group (TMG): The TMG monitored all aspects of the conduct and progress of the trial, ensured that the protocol is adhered to and take appropriate action to safeguard participants and the quality of the trial itself. The TMG included the Chief Investigator (N.S.), trial manager (K.J.M.B.) and representatives from the Glasgow Clinical Trials Unit (GCTU), NHS GG&C and the University of Glasgow.

The NHS Sponsor monitored the trial. Since previous studies indicate that the study drug is well tolerated in this patient group, there was no Independent Data and Safety Monitoring Committee (IDMC).

Supplemental Results

Safety Outcomes

There were no between-group differences in safety outcomes (Supplemental Table 3). Specifically, there were no cases of diabetic ketoacidosis and no lower limb amputations. There were 2 deaths in the empagliflozin group, one due to newly diagnosed pancreatic cancer and one due to cardiogenic shock.

Exploratory Outcomes

Body weight decreased by 1.4 kg between baseline and 36 weeks in the empagliflozin group compared to an increase of 0.4 kg in the placebo group: adjusted between-group difference -1.9 (95% CI -3.6 to -0.2) kg; p=0.031 (Supplemental Table 4). There were no significant changes in systolic and diastolic blood pressure, heart rate, or blood ketones after 36 weeks of treatment with empagliflozin, compared to placebo (Supplemental Table 4).

Supplemental Tables and Figures Legends

Supplemental Table 1. Schedule of Study Visits
Supplemental Table 2. Typical Imaging Acquisition Parameters for CMR
Supplemental Table 3. Safety Outcomes: Number of subjects that experienced at least one of the following safety outcomes
Supplemental Table 4. Change in Exploratory Outcomes with Empagliflozin 10mg/day or Placebo from Baseline to Week 36
Supplemental Figure 1. CONSORT diagram

Supplemental Table 1. Schedule of Study Visits

Study Procedure	Screening Visit (0-12 weeks or 0-84 days before Visit 1)	Baseline Visit 1 (Week 0)	Visit 2 (Week 2 ± 3 days)	Visit 3 (Week 12 ± 2 weeks)	Phone Call (Week 24 ± 2 weeks)	Visit 4 (Week 36 ± 4 weeks)	Visit 5 (Week 40 ± 4 weeks)
Obtain informed consent	✓						
Randomization		✓					
Review inclusion and exclusion criteria		✓					
Pregnancy test in women of childbearing potential		✓		✓		✓	✓
Basic demographic and past medical history questionnaire		✓					
Medication history		✓	✓	✓		✓	✓
Physical examination		✓	✓	✓		✓	✓
Vital signs (blood pressure, pulse)		✓	✓	✓		✓	✓
Height (Week 0 only), weight		✓	✓	✓		✓	✓
Bioelectrical impedance analysis		✓	✓	✓		✓	✓
6 minute walk test		✓		✓		✓	
Kansas City Cardiomyopathy Questionnaire (KCCQ)		✓		✓		✓	✓
Michigan Neuropathy Screening Instrument (MNSI)		✓				✓	
Biobanking & laboratory tests	✓ (screening)	✓	✓	✓		✓	✓
Blood glucose & ketones		✓	✓	✓		✓	✓
Standard electrocardiogram		✓		✓		✓	✓
Echocardiogram	✓					✓	
Lung ultrasound		✓		✓		✓	✓
MRI (cardiac and renal)		✓				✓	✓
Review of AESIs/SAEs and safety questions			✓	✓	✓	✓	✓
Pill count			✓	✓		✓	
Dispensing of study drug		✓		✓			

Abbreviations: AESIs, adverse events of special interest; SAE, serious adverse events.

Supplemental Table 2. Typical Imaging Acquisition Parameters for CMR

Parameter	Cine	T1 map	Cardiac rest perfusion (DCE)
**Orientation**	VLA, HLA, LVOT, SAX stack	SAX mid LV, HLA, Transverse AIF*	Transverse AIF*, SAX mid LV
**Sequence**	CS	MOLLI	SR-TurboFLASH
**Breath-hold**	Breath-hold	Breath-hold	Gentle breathing
**TR, ms**	52.02	Pre-contrast: 280.56<br>Post-contrast: 360.56	167.00
**TE, ms**	1.23	1.12	0.98
**TI, ms**	N/A	Pre-contrast: 180<br>Post-contrast: 260	90
**Flip angle, °**	50	35	10
**FOV, mm*mm**	287.69x340.00	306.56x360.00	360.00x360.00
**Matrix**	176x208	218x256	192x192
**Slice thickness (mm)**	7	8	8
**Slice gap (mm)**	3	n/a	n/a
**Number of slices**	1 VLA<br>1 HLA<br>1 LVOT<br>SAX stack	1 mid SAX<br>1 HLA<br>1 AIF	1 AIF<br>1 mid SAX
**Acceleration**	N/A (CS)	GRAPPA - 2	GRAPPA - 2
**Total acquisition time (min:sec)**	12:00	05:00	15:00
**Bandwidth (Hz/px)**	962	1085	1184
**No. of preps**	N/A	Pre-contrast: 2<br>Post-contrast: 3	N/A
**Sampling duration**	N/A	Pre-contrast: 5 beats<br>Post-contrast: 4 beats	N/A
**Recovery duration**	N/A	Pre-contrast: 3 beats<br>Post-contrast: 1 beat	N/A

* Transverse aortic plane at the level of the main pulmonary artery.
Abbreviations: AIF, arterial input function; CMR, cardiovascular magnetic resonance; CS, compressed sensing; DCE, dynamic contrast-enhanced; FOV, field of view; GRAPPA, GeneRalized Autocalibrating Partial Parallel Acquisition; HLA, horizontal long axis; LGE, late gadolinium enhancement; LV, left ventricle; LVOT, left ventricular outflow tract; MOLLI, modified Look-Locker inversion-recovery; N/A, not applicable; SAX, short axis; SR-TurboFLASH, saturation recovery Turbo Fast Low-Angle Shot; SSFP, steady-state free precession; TE, echo time; TI, inversion time; TR, repetition time; VLA, vertical long axis.

Supplemental Table 3. Safety Outcomes: Number of subjects that experienced at least one of the following safety outcomes

Safety Outcomes, N (%)	Empagliflozin (n=52)	Placebo (n=53)	*P* Value*
**Safety Assessments (Weeks 2, 12, 24 (phone), 36 and 40)†**			
Hepatic injury‡	0 (0.0%)	0 (0.0%)	-
Renal dysfunction / hyperkalemia / metabolic acidosis‡	6 (11.5%)	9 (17.0%)	0.58
Ketoacidosis‡	0 (0.0%)	0 (0.0%)	-
Any symptoms of hypotension/volume depletion including presyncope/syncope and falls (and measured blood pressure)	29 (55.8%)	31 (58.5%)	0.84
Any urinary tract infection episodes	7 (13.5%)	5 (9.4%)	0.56
Any genital infection	7 (13.5%)	4 (7.5%)	0.36
**Safety Assessments (Weeks 2, 12, 36 and 40)**			
Creatinine levels > 221 micromol/l	1 (1.9%)	1 (1.9%)	1.00
**Serious Adverse Events (Baseline to Week 40 + 30 Days)§**			
Any event	15 (28.8%)	16 (30.2%)	1.000
**Cardiac disorders**	10 (19.2%)	6 (11.3%)	0.290
Acute myocardial infarction	3 (5.8%)	1 (1.9%)	
Cardiac failure	3 (5.8%)	0 (0.0%)	
Atrial fibrillation	1 (1.9%)	1 (1.9%)	
Acute coronary syndrome	0 (0.0%)	1 (1.9%)	
Angina pectoris	0 (0.0%)	1 (1.9%)	
Angina unstable	0 (0.0%)	1 (1.9%)	
Cardiac arrest	0 (0.0%)	1 (1.9%)	
Cardiac failure acute	1 (1.9%)	0 (0.0%)	
Cardiogenic shock	1 (1.9%)	0 (0.0%)	
Myocardial infarction	0 (0.0%)	1 (1.9%)	
Palpitations	1 (1.9%)	0 (0.0%)	
Sinus node dysfunction	1 (1.9%)	0 (0.0%)	
**Metabolism and nutrition disorders**	4 (7.7%)	5 (9.4%)	1.000
Hyperkalemia	4 (7.7%)	5 (9.4%)	
**Infections and infestations**	2 (3.8%)	2 (3.8%)	1.000
Abscess limb	1 (1.9%)	0 (0.0%)	
Biliary sepsis	0 (0.0%)	1 (1.9%)	
Influenza	0 (0.0%)	1 (1.9%)	
Lower respiratory tract infection	1 (1.9%)	0 (0.0%)	
Pneumonia	1 (1.9%)	0 (0.0%)	
**Injury, poisoning and procedural complications**	1 (1.9%)	2 (3.8%)	1.000
Alcohol poisoning	0 (0.0%)	1 (1.9%)	
Femoral neck fracture	1 (1.9%)	0 (0.0%)	
Head injury	0 (0.0%)	1 (1.9%)	
**Gastrointestinal disorders**	0 (0.0%)	2 (3.8%)	0.495
Gastroesophageal reflux disease	0 (0.0%)	1 (1.9%)	
Rectal hemorrhage	0 (0.0%)	1 (1.9%)	
**Hepatobiliary disorders**	1 (1.9%)	1 (1.9%)	1.000

Cholecystitis acute	0 (0.0%)	1 (1.9%)	
Liver injury	1 (1.9%)	0 (0.0%)	
**Eye disorders**	0 (0.0%)	1 (1.9%)	1.000
Retinal artery occlusion	0 (0.0%)	1 (1.9%)	
**General disorders and administration site conditions**	1 (1.9%)	0 (0.0%)	0.495
Chest pain	1 (1.9%)	0 (0.0%)	
**Neoplasms benign, malignant and unspecified (incl cysts and polyps)**	1 (1.9%)	0 (0.0%)	0.495
Pancreatic carcinoma metastatic	1 (1.9%)	0 (0.0%)	
**Renal and urinary disorders**	1 (1.9%)	0 (0.0%)	0.495
Acute kidney injury	1 (1.9%)	0 (0.0%)	
**Vascular disorders**	0 (0.0%)	1 (1.9%)	1.000
Peripheral artery aneurysm	0 (0.0%)	1 (1.9%)	

* *P* values taken from Fisher's test; † Safety Assessments were completed at weeks 2, 12, 24 (phone), 36 and 40 and therefore, one hepatic injury event that occurred after week 2 requiring discontinuation of study drug was not recorded here because that subject did not return for further visits; ‡ Adverse Event of Special Interest (AESI); § Serious Adverse Events summarized and sorted by Medical Dictionary for Regulatory Activities (MedDRA) System Organ Class (SOC) term order and by preferred term order within SOCs. There was no between-group difference in the total number of Serious Adverse Events (23 in empagliflozin group, 22 in placebo group, p=0.95 using Wilcoxon test to compare the number of Serious Adverse Events per person in each group). One myocardial infarction event in the placebo group was classed as a "related" Serious Adverse Event and was not included in table above.

Supplemental Table 4. Change in Exploratory Outcomes with Empagliflozin 10mg/day or Placebo from Baseline to Week 36

Variable*	Empagliflozin				Placebo				Between group difference (95% CI)†	*P* Value
	N	Baseline	Week 36	Change	N	Baseline	Week 36	Change		
**Exploratory Outcomes**										
Systolic BP, mmHg	44	127.1 (18.9)	124.8 (18.0)	-2.4 (13.8)	50	128.8 (21.3)	128.3 (18.9)	-0.5 (15.9)	-1.9 (-7.5, 3.6)	0.49
Diastolic BP, mmHg	44	71.1 (10.4)	71.2 (11.3)	0.1 (11.1)	50	71.6 (12.8)	71.2 (10.9)	-0.4 (11.2)	0.2 (-3.6, 4.1)	0.90
Heart rate, bpm	44	69.7 (12.7)	66.2 (10.8)	-3.5 (11.2)	50	64.3 (9.2)	62.4 (8.7)	-1.9 (7.6)	0.4 (-2.9, 3.7)	0.82
Body weight, kg	44	85.2 (19.1)	83.9 (20.2)	-1.4 (4.0)	50	86.5 (15.2)	87.0 (14.6)	0.4 (4.4)	-1.9 (-3.6, -0.2)	0.031
Blood ketone, mmol/l	44	0.18 (0.07)	0.22 (0.09)	0.03 (0.10)	50	0.17 (0.09)	0.19 (0.08)	0.02 (0.11)	0.025 (-0.0008, 0.058)	0.14

* Mean (SD); † Treatment effect calculated using an ANCOVA model adjusted for the treatment group, age at baseline, diabetes status, and baseline value of the outcome.

Abbreviations: BP, blood pressure.

Supplemental Figure 1. CONSORT diagram

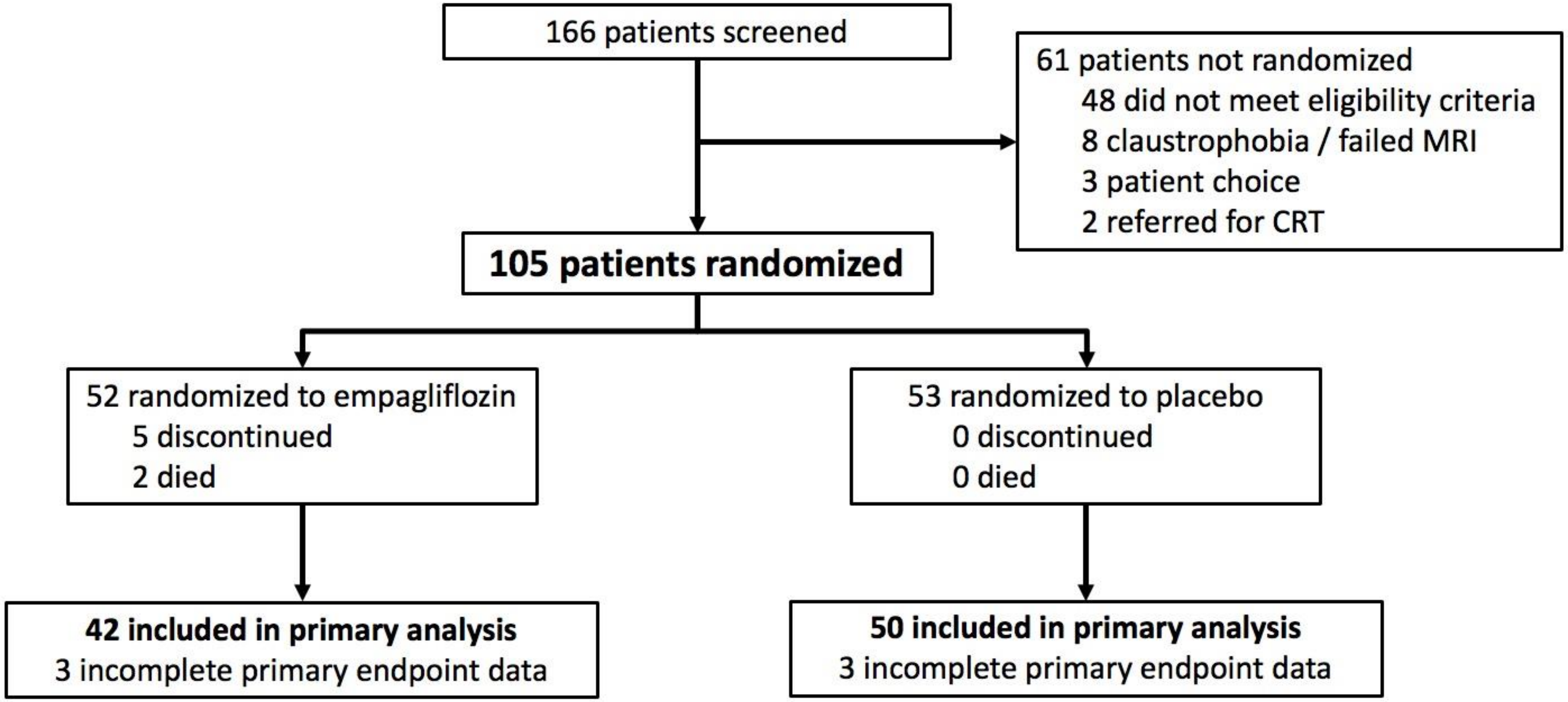

All 5 patients who discontinued empagliflozin did so prematurely with < 3 months of cumulative drug exposure, and therefore did not attend for follow-up MRI as per study protocol. The reasons for discontinuation of study medication were: 3 nausea and vomiting, 1 weight loss, 1 hepatic injury (adverse event of special interest necessitating permanent withdrawal of study medication as per protocol). There were 2 deaths in the empagliflozin group: 1 newly diagnosed pancreatic cancer, 1 cardiogenic shock. Of the 6 patients with incomplete primary endpoint data at week 36: 3 in atrial fibrillation or atrial flutter at week 36 and were excluded from primary analysis, 1 unanalyzable week 36 MRI images (major artefacts), 1 MRI contraindication at week 36 (silver foot dressing), 1 did not attend week 36 visit due to pandemic (coronavirus disease 2019).

Acknowledgements
We are enormously grateful to the SUGAR-DM-HF trial volunteers for their participation.

Research Nurses – Ann Wright, Joyce Thompson, Barbara Meyer

Clinical Research Imaging Facility – Tracey Hopkins, Evonne McLennan, Laura Dymock, Fiona Savage, Nicola Tynan, Patricia Clarkin, Sarah Allwood-Spiers, Pauline Hall Barrientos

University of Glasgow Biomarker Laboratory – Philip Stewart, Elaine Butler, Emma Dunning

Robertson Centre for Biostatistics – Mairi Warren, Sharon Kean, David Jamieson, Ross Clarke, Robbie Wilson

Independent members of Trial Steering Committee – Robbie Lindsay (chair), Rachel Myles, Rajan Patel

Pharmacy – Elizabeth Douglas, Helen Hart, Karen Montgomery, Doreen Ritchie, Colin Rodden, Samantha Carmichael, Pamela Surtees

Sponsor Representative (NHS Greater Glasgow and Clyde) – Maureen Travers

Pharmacovigilance – Marc Jones

Monitoring – Sheila McGowan, Temitope Ibitoye, Lyndsay McDade, Barbara Ross

Independent advisor (named on patient information sheet) – Gemma Currie

MRI Support (Siemens Healthineers) – Peter Weale

MRI Support (circle cvi42) – Donatella Lorusso, Jennifer Mancio, Sam Meynell

University of Glasgow British Heart Foundation Glasgow Cardiovascular Research Centre – Christopher Rush, Peter McCartney

Clinical teams who supported trial recruitment and ongoing clinical care of trial patients:

- Queen Elizabeth University Hospital – Faheem Ahmed, Colin Berry, John Byrne, James Cairns, Ross Campbell, Andrew Davie, Kieran Docherty, Pardeep Jhund, Ninian Lang, David McGrane, John McMurray, Kirstie Mowat, David Murdoch, Piotr Sonecki, Pernille Sorensen
- Glasgow Royal Infirmary and Stobhill Hospital – Mark Petrie (Principal Investigator), Fiona Bell, James Boyle, John Carson, JulieAnn Earley, Miles Fisher, Matthew Lee, Joanne Lindsay, John Petrie, Lynda Robertson, Kirstin Russell, Jackie Taylor
- West Glasgow Ambulatory Care Hospital – Iain Speirits, Neil McInnes, Kirstie Mowat, Valerie O’Neill
- New Victoria Hospital – Jane Cannon, Colette Jackson, Howard McAlpine, Katriona Mullen, Claire O’Neill, Eve Shannon
- Royal Alexandra Hospital and Vale of Leven Hospital – Clare Murphy, Aylison Clark, Alistair Cormack, Eric Gray, Stuart Hood, Linda McGinnis, Maureen Melvin, Eileen Peat, Keith Robertson

- Inverclyde Royal Hospital – Lorraine Carey-Gardner, Omar Hasan, Andrew McCulloch, Helen Papaconstantinou, Sheila Skinner
- University Hospital Monklands – Colin Petrie (Principal Investigator), Andrea Lees
- University Hospital Wishaw – Karen Gilmour, Louise Gray, Siaw Cheng Teo, Caroline White
- University Hospital Hairmyres – Jacquie Baird, Claire Millarvie, Robin Weir
- University Hospital Crosshouse – Victor Chong (Principal Investigator), Fiona McAskill, Janet McKay, Gillian Mitchell
- University Hospital Ayr – Vera Lennie (Principal Investigator)
- Forth Valley Royal Hospital – Catherine Labinjoh (Principal Investigator), Jennifer Flanagan, Kerrie McMenemy, Linda McShane, Catherine Mondoa, Gareth Padfield, Nicola Wilson, Sowmya Venkatasubramaniam
- Golden Jubilee National Hospital – Mark Petrie (Principal Investigator), Roy Gardner
